# The Formation and Change of Volatile Flavor Compounds During the Cooking of Sheep Bone Soup

**DOI:** 10.3390/foods14060949

**Published:** 2025-03-11

**Authors:** Shan Wu, Yuzhu Bai, Baocai Xu, Xinfu Li, Zhong Yao, Jingjun Li, Yun Sun

**Affiliations:** 1 College of Food Science and Light Industry, Nanjing Tech University, Nanjing 211816, China; 2 School of Food and Biological Engineering, Hefei University of Technology, Hefei 230009, China; 3 College of Food Engineering, Anhui Science and Technology University, Chuzhou 239000, China

**Keywords:** sheep bone soup, TBARS value, fatty acids, Maillard reaction, volatile flavor compounds

## Abstract

To investigate the formation of flavor compounds in sheep bone soup, E-nose, gas chromatograph (GC), and gas chromatography-mass spectrometry (GC-MS) were used to determine the changes in lipid oxidation, Maillard reaction, and volatile flavor compounds during the slow cooking process of 4 h. The thiobarbituric acid reactive substances (TBARS) value began to increase significantly (*p* < 0.05) after 2 h of cooking, reaching its peak in the third hour before significantly decreasing. The intensity of the Maillard reaction significantly increased after 2 h of cooking and subsequently stabilized. Thirty-nine flavor compounds were identified, primarily comprising aldehydes, ketones, alcohols, esters, aromatic compounds, and heterocyclic compounds. The formation of volatile flavor compounds in sheep bone soup was associated with lipid oxidation, particularly the oxidation of unsaturated fatty acids, and the Maillard reaction. Lipid oxidation produced a large number of volatile flavor compounds, such as aldehydes and ketones. The Maillard reaction gave sheep bone soup a certain flavor. Aldehydes were mostly influenced by cooking time, becoming the main flavor compounds in the bone soup after 2.5 h of cooking, accounting for more than half of the total volatile flavor compounds. The highest content and richest profile of volatile flavor compounds were obtained in the soup cooked for 2.5 h and 3 h. This study provides a theoretical basis for the flavor regulation of sheep bone soup.

## 1. Introduction

Bone soup is prepared by cooking bones in water. With abundant nutrients, including collagen, fats, calcium, and vitamins, and a uniquely delightful flavor [1], bone soup not only serves as a food item but can also function as a condiment to enhance the flavor of various foods and is popular in Eastern culinary cultures. Sheep bone soup is known for its warm nature recorded in Chinese medicine and delicious flavor and is believed to be able to strengthen bones and warm the bodies of the people who consume sheep bone soup, making it particularly well-received in China’s cold and humid regions [2]. The unique flavors are important for the quality and acceptability of bone soup. Meng et al. [3] found that during the cooking process, the dissolution of nutrients from the bones was accompanied by changes in the composition of volatile flavor compounds. The formation of volatile flavor compounds during cooking is essential for the quality control of bone soup.

Flavor compounds are primarily formed through lipid oxidation, Maillard reaction, amino acid degradation, and thermal degradation of thiamine during food processing [4]. Lipid oxidation and Maillard reaction are recognized as the most important pathways [5]. The oxidation of lipid acyl chains accounts for a substantial part of lipid oxidation. Meng et al. [3] found that the flavor compounds in beef bone soup, aldehydes and ketones, can be derived from lipid oxidative degradation. Qi et al. [6] concluded that aldehydes were mainly derived from lipid oxidation and degradation, and hexanal significantly impacted the flavor of chicken soup. Xie et al. [7] hypothesized that oleic acid and linoleic acid might play a critical role in the formation of aldehydes and ketones in thermally processed water-boiled salted duck. Maillard reaction, also known as the amino-carbonyl reaction, is also important for the generation of flavor in heat-processed foods. Wang et al. [8] found that the main factor influencing the development of tea flavor was the Maillard reaction that took place when the tea leaves were drying. Zhang et al. [9] believed that the flavor formation during the cooking of pork bone soup was related to the Maillard reaction, which led to the formation of volatile compounds such as heterocyclic compounds.

The flavor of bone soup is affected by pressure, temperature, and time used in the cooking process. High-pressure cooking (121 °C) and atmospheric-pressure cooking (98–100 °C) are common heating methods used in soup processing. Sang et al. [10] found that high-pressure conditions could shorten the heat time, but the color and flavor of bone soup cooked at high pressure were relatively poor compared with that cooked at atmospheric pressure. Qin et al. [11] found that eight additional types of volatile flavor compounds were detected in high-pressure chicken skeleton soup compared to atmospheric pressure, with higher relative contents of hydrocarbons and esters, while ketones were lower. The beef soup prepared at lower temperatures (85–95 °C) exhibited superior flavor and quality compared to that cooked at higher temperatures (above 95 °C) [12]. Chotechuang et al. [13] examined the free amino acid content and sensory quality of chicken bone soup cooked at 85 °C, 90 °C, and 95 °C for different periods of time (30, 60, 120, and 180 min), the results showed that the chicken bone soup heated at 90–95 °C for 180 min had better taste and flavor quality. Qi et al. [6] obtained chicken soup with both fresh flavor and aroma after 2 h of cooking at 95–99 °C. Meng et al. [3] identified the effects of cooking time on the quality of bovine bone for 10 h and found that prolonged heating facilitated nutrient dissolution but destroyed the diversity of flavor compounds, leading to an overall loss of volatile flavors.

Prior research on sheep bone soup has predominantly focused on enhancing nutrient dissolution and improving sensory properties through enzymatic hydrolysis [14] and optimizing cooking conditions, such as time and bone–water ratio [15]. The boiling time varies according to the researchers’ objectives, ranging from less than one hour to six hours [1]. However, there is still a lack of research on the formation and change in volatile flavors during sheep bone soup cooking, making it difficult to control the flavor and quality of the product. In this study, the changes in volatile flavor compounds, lipid oxidation, and the Maillard reaction related to flavor formation during atmospheric pressure cooking of pure sheep bone soup were investigated, which can provide a reference for the control of flavor quality and process optimization of sheep bone soup.

## 2. Materials and Methods

### 2.1. Materials

Fresh sheep bones were purchased from Lu’an Cuisine Fresh Food Co., Ltd. (Lu’an, China), weighing 150 ± 20 g and including proximal compositions. Sheep bones were transported by cold chain at −18 °C and stored at −18 °C. A total of 37 kinds of fatty acid methyl ester mixed standards (C7–C30) were purchased from Shanghai ANPEL-TRACE Technical Service Co., Ltd. (Shanghai, China). In addition, 1,2-dichlorobenzene used for gas chromatography-mass spectrometry (GC-MS) was purchased from Sigma-Aldrich Co., Ltd. (Shanghai, China). All other reagents were analytically pure and were purchased from Sinopharm Chemical Reagent Co., Ltd. (Shanghai, China).

### 2.2. Sample Preparation

Frozen sheep bones were thawed using flowing water, washed until the water ran clear, and then broken into blocks of 6 cm × 3 cm × 2 cm. The bones were then pre-cooked for 5 min to remove blood stains and other impurities. Bones were mixed with purified water at a 1:3 ratio (by weight) and boiled at eight time gradients (0.5 h, 1 h, 1.5 h, 2 h, 2.5 h, 3 h, 3.5 h, and 4 h), respectively; each cooking time point (0.5 h, 1 h, etc.) was repeated three times. The induction cooker was initially set to 1000 W until the water boiled and then reduced to 300–500 W to maintain a slight boil. The boiling temperature fluctuated within the range of 99.4–100.2 °C. At the end of boiling, the bone soup samples were filtered with gauze and stored at −80 °C to be tested. Portions of the sheep bone soup samples were concentrated to five times their original concentration using a rotary evaporator under vacuum, with a vacuum degree of −0.08 MPa, rotational speed of 95 rpm, and temperature of 55 °C.

### 2.3. Determination of TBARS Value

The TBARS value was measured according to the method of Veberg et al. [16], with some modifications. A total of 10 mL of concentrated bone soup sample was added to 50 mL of 7.5% trichloroacetic acid (containing 0.1% EDTA) and shaken for 30 min at 150 rpm, followed by filtration through double-layer filter paper. Then, 10 mL of supernatant was transferred into a centrifugal tube, followed by the addition of 10 mL thiobarbituric acid (TBA) reagent. The mixture was incubated at 90 °C for 50 min using a water bath and cooled to room temperature. Then, combined with 10 mL chloroform, the samples were vortexed to ensure complete mixing, followed by phase separation under static conditions. Then, the supernatant was collected for spectrophotometric analysis at wavelengths of 532 nm and 600 nm. The maximum absorbance was measured at 532 nm, and the interference value was measured at 600 nm. The TBARS value was calculated using a standard curve of malonaldehyde (MDA).

### 2.4. Determination of the Degree of Maillard Reaction

A total of 10 mL of concentrated sheep bone soup was mixed with 10 mL of pre-cooled 20% (*v*/*v*) trichloroacetic acid (TCA). The mixture was centrifuged at 7000 rpm for 10 min at 4 °C, and the supernatant was filtered using filter paper. Maillard reaction intermediates were quantified by measuring the absorbance at 280 nm, while the browning intensity was assessed via absorption at 420 nm. Another 1 mL of the concentrated sample of sheep bone soup was added to 4 mL of dichloromethane–ethanol (2:1, *v*/*v*) and 0.1 mM butylated hydroxytoluene as an antioxidant, stirred for 15 min, and then centrifuged (6605 rpm, 15 min). The fluorescence intensity (FI) of the supernatant was measured after stratification. The fluorescence excitation wavelength was 360 nm, with an excitation slit of 10.0 nm and an emission wavelength of 390–600 nm [17].

### 2.5. Fatty Acids Analysis

Taking 30 mL of concentrated sheep bone soup sample, 180 mL of chloroform–methanol solution (2:1, *v*/*v*) was added and filtered after shaking for 2 h at 45 °C. A total of 50 mL of saturated NaCl solution was added to the filtrate, shaken, and allowed to stratify. The filtrate was concentrated by a rotary evaporator in a water bath at 45 °C to obtain the lipid, which was weighed accurately after drying. Then, 5 mL of the mixture of benzene and petroleum ether (1:1, *v*/*v*) was added to the dried lipid, and the lipid was dissolved by shaking. Then, 3 mL of 14% boron trifluoride–methanol solution was added and mixed thoroughly. The reaction was carried out in a water bath at 45 °C for 30 min. A total of 1 mL of hexane solution was added to dissolve the methyl ester, and then an appropriate amount of saturated NaCl solution was added to make all of the organic phase methyl ester solutions rise to the upper part of the test tube. The supernatant was sucked up and added to 1 mL of 0.55 mg/mL methyl heptadecanoate, then the solvent was dried using a nitrogen-blowing instrument and added to 1 mL of n-hexane (chromatographic grade) to adjust the final volume for optimal GC injection.

The gas chromatograph (Agilent 7890b) was equipped with an SP-2560 capillary column (100 m × 0.25 mm × 0.20 μm). The parameters of the running program were referred to Xie et al. [7]. Qualitative analysis was carried out by comparing the retention times of the sample peaks with that of 37 fatty acid mixture standards. Fatty acid quantification was conducted through the internal standard method, with methyl heptadecanoate serving as the reference marker.

### 2.6. E-Nose Analysis

E-nose analysis was used to investigate the flavor profile of sheep bone soup. The E-nose (PEN3, AIRSENSE Co., Schwerin, Germany) was equipped with 10 metal sensors. A 5 mL sample of sheep bone soup was added into a 20 mL headspace bottle and equilibrated at 60 °C for 20 min. The analysis parameters of the E-nose were set as follows: the cleaning time was 100 s, zero time was 10 s, pre-sampling time was 5 s; the headspace injection volume was 400 μL, the injection speed was 400 μL/s; the sample collection time and cycle time were 120 s and 1 s, respectively. The carrier gas of the electronic nose was high-purity air, the flow rate was 400 mL/min, and the determination of each sample was performed in triplicate.

### 2.7. Analysis of Volatile Compounds by Gas Chromatography-Mass Spectrometry (GC-MS)

The analysis referred to the method of Liu et al. [18], with some modifications. A gas chromatography-mass spectrometry (GC-MS) instrument (8890-5977B, Agilent Co., Santa Clara, CA, USA) was used to determine the volatile compounds in sheep bone soup. The GC-MS was equipped with an automatic headspace injection system and an HP-5MS column. Five mL of sheep bone soup was added to a 20 mL headspace bottle, equilibrated at 60 °C in a water bath for 20 min, and then an SPME fiber was inserted into the bottle to adsorb for 40 min, which was previously purged with nitrogen and aged for 30 min at 250 °C in the injection portAfter extraction, the SPME fiber was injected into the injection port to desorb the extracted compounds for 10 min. The operating procedure of the instrument was as follows: the flow rate was 1 mL/min, the inlet temperature was 250 °C, and the carrier gas was high-purity nitrogen. The heating procedure was set as follows: the column temperature was initially maintained at 40 °C for 3 min, then increased by 5 °C/min to 90 °C, then increased to 230 °C at 10 °C/min and held for 8 min. An electron ionization (EI) source was used: the electron energy was 70 eV, the ion source and the interface temperatures were 200 °C and 250 °C, respectively, and the scanning mass range was 45–500 *m*/*z*.

The mass spectra of volatile flavor compounds were identified by matching with those in the NIST 20. L library (National Institute of Standards and Technology, Gaithersburg, MD, USA) and confirmed by retention indices (RIs). Quantitative analyses were performed by comparing the peak area of volatile compounds with the internal standard peak area using 1,2-dichlorobenzene as an internal standard.

### 2.8. Statistical Analysis

One-way analysis of variance (ANOVA) and Waller–Duncan homogeneity tests were conducted using SPSS Statistics 23.0 software to assess the significant differences between samples from different sampling times (*p* < 0.05). Principal component analysis (PCA) and partial least squares discriminant analysis (PLS-DA) were performed using MetaboAnalyst 6.0. The determinations of samples from different sampling times were performed in three parallel groups, and the data results were presented as the mean ± standard deviation.

## 3. Results and Discussion

### 3.1. Changes in TBARS Value During the Cooking of Sheep Bone Soup

Moderate lipid oxidation is an important pathway for the formation of volatile flavor compounds in meat products [19]. The TBARS value can reflect the degree of lipid oxidation by determining the amount of the lipid secondary oxidation product MDA [20].

**Figure 1:**
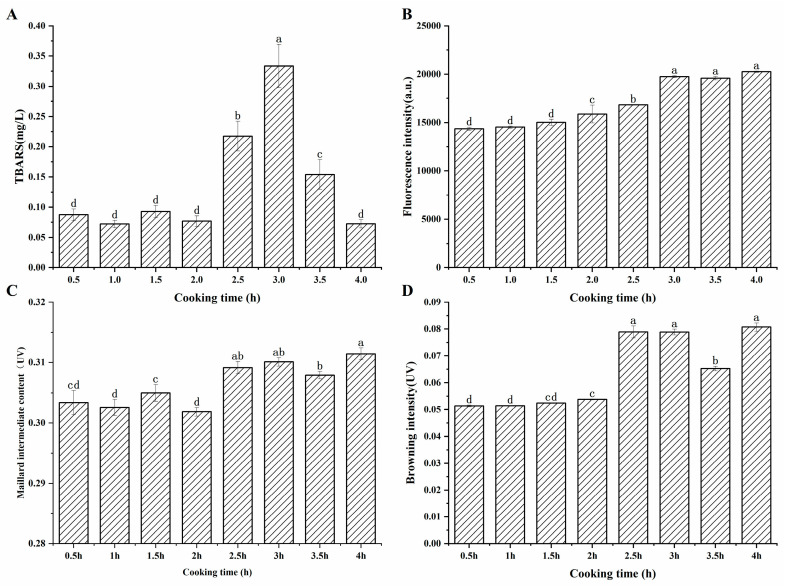
(**A**), Changes in TBARS values in sheep bone soup during cooking (**B**), fluorescence intensity (**C**), content of Maillard intermediates (**D**), browning intensity. ^a–d^ represent significant differences (*p* < 0.05) between samples at different cooking times.

According to Figure 1A, early in the cooking process, the TBARS value was generally low, and the change was not significant (*p* > 0.05). After two hours of cooking, the TBARS value began to rise significantly (*p* < 0.05), reaching its peak at 3 h, and then significantly decreasing as the cooking time was prolonged. The increase in TBARS value was attributed to the increase in the degree of lipid oxidation [21], leading to the accumulation of MDA, after a long period of heating. Simultaneously, MDA was prone to reacting with other components such as proteins, phospholipids, and amino acids, resulting in the loss of MDA [22]. Moreover, another reason for the reduction in TBARS content may be that aldehydes can be further oxidized to organic alcohols and carboxylic acids [23]. In addition, active carbonyl compounds, such as dialdehydes and unsaturated aldehydes generated by lipid oxidation, can participate in the advanced stages of the Maillard reaction [24]. The reduction in the TBARS value three hours later suggested that the reactive loss of MDA took the leading position.

### 3.2. Changes in the Degree of Maillard Reaction During the Cooking Process of Sheep Bone Soup

Changes in fluorescence intensity, Maillard intermediate content, and browning intensity can be a comprehensive response to the extent of the Maillard reaction [25]. Fluorescence intensity reflects the early stage of the Maillard reaction, and there are two pathways for the formation of fluorescent pigments during the heating process: one is the oxidation reaction between proteins and the aldehyde products of lipid peroxidation, and the other is the reaction of proteins with reducing sugars [17]. The Maillard intermediate content indicates the amount of colorless products formed in the middle process of the Maillard reaction, such as sugars, aldehydes, and small-molecule ketones [26]. Browning intensity correlates with brown polymer generation during the terminal Maillard reaction phase, directly influencing product coloration [27].

Figure 1B exhibited that the fluorescence intensity at the maximum absorption wavelength of 414 nm showed a gradually increasing trend, and the change was significant after 1.5 h of cooking (*p* < 0.05). Veberg et al. [16] added aldehydes to minced meat in their experiment, and the maximum absorption peak was observed at 410–550 nm, which suggested that the fluorescent pigments may be produced by the reaction of unsaturated aldehydes with proteins. It can be found that after cooking for 3 h, the fluorescence intensity remained at a relatively high level. As seen in Figure 1C, the content of the intermediate products of the Maillard reaction fluctuated during the first two hours of cooking, then increased significantly and remained essentially unchanged. Maillard intermediates were formed and simultaneously consumed during the heating process. This complex phenomenon may be related to the formation and degradation of sugar, Strecker degradation, and additional involvement of the carbonyl ammonium reaction during the Maillard reaction [28]. The dynamic reaction caused variations in the content of Maillard intermediates. Figure 1D showed that the browning degree displayed a continuous upward trend and increased significantly after 2 h of cooking, which may be attributed to the prolonged heating that promoted the process of the Maillard reaction and the accumulation of brownish substances.

### 3.3. Changes in Fatty Acid Contents During the Cooking Process of Sheep Bone Soup

The lipid profile and the content of fatty acids in meat products impact their quality characteristics, including flavor and aromatic taste profile [29]. The determination of fatty acids has been used to investigate the alterations induced by lipid oxidation and hydrolysis [30].

A total of 14 fatty acids were detected in sheep bone soup at different cooking times, including 9 saturated fatty acids, 3 monounsaturated fatty acids, and 2 polyunsaturated fatty acids. Saturated fatty acids accounted for a higher proportion of fatty acids in sheep bone soup, and the proportion increased from 59% at the start to 91% by the end of the cooking process, which was dominated by C8:0 (octanoic acid), C11:0 (undecanoic acid), and C18:0 (stearic acid). Unsaturated fatty acids mostly consisted of C14:1 (myristoleic acid), C16:1 (palmitoleic acid), and C18:1 (oleic acid). The content of monounsaturated fatty acids (MUFA) is positively correlated with food flavor and overall acceptability according to the report of Cambero et al. [12].

Table 1 showed that the composition and content of fatty acids changed throughout the cooking process. With the gradual release and oxidative decomposition of fatty acids during cooking, the content of saturated fatty acids generally showed a fluctuating upward trend. However, the content of unsaturated fatty acids demonstrated a fluctuating downward tendency, indicating that fatty acids were released while concurrently undergoing oxidative decomposition loss during the cooking process of sheep bone soup, especially unsaturated fatty acids, which were more susceptible to oxidative degradation, resulting in a fluctuating decrease in their content. By comparing with the changes in TBARS values (Figure 1A), it can be inferred that the significant increase in TBARS value after cooking for 2 h was attributed to the oxidative degradation of fatty acids, especially unsaturated fatty acids during the cooking process of sheep bone soup.

Among unsaturated fatty acids, the content and changes in oleic acid are the most typical. Oleic acid is an important flavor fatty acid among monounsaturated fatty acids, Choe et al. [31] believed that oleic acid is closely related to meat flavor. The content of oleic acid decreased significantly (*p* < 0.05) during cooking, probably caused by the continuous oxidation of the double bonds of unsaturated fatty acids under prolonged high-temperature cooking, which led to a decrease in unsaturation and degradation to produce different volatile aldehydes, ketones, and lower hydrocarbons [32].

### 3.4. Changes in E-Nose Data During the Cooking Process of Sheep Bone Soup

**Figure 2:**
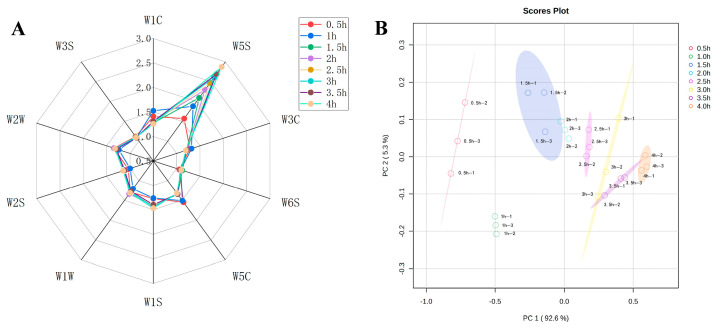
The date of E-nose measurements at different cooking times (**A**), radar chart of the E-nose sensors (**B**), principal component analysis biplot.

Table 2 showed the specific information of each sensor of the E-nose. The flavor composition of the sheep bone soup was determined by calculating the sensitivity substances corresponding to the 10 sensors. The higher the response values of the sensors, the greater the variation in the response values and the better the differentiation effect on volatile odors [33]. To analyze the flavor changes in sheep bone soup during cooking, a radar plot was constructed of the response values of the 10 different sensors to the odors. The variations in volatile compound profiles under different cooking times were predominantly captured by sensors W5S, W1C, W5C, and W2W (Figure 2A), suggesting that the key flavor characteristics of sheep bone soup can be attributed to nitrogen oxides, benzene, short-chain alkanes, and organic sulfide. Furthermore, the differences between the response values of sensor W5S were more obvious.

Principal component analysis was applied to further examine the electronic nose analysis data of sheep bone soup. The contribution rates of PC1 and PC2 were shown in Figure 2B to be 92.6% and 5.3%, respectively, which meant that the first principal component can reflect the original information of the whole sample. The total contribution rate reached almost 98%, indicating that the two principal components could reflect the overall flavor information of sheep bone soup samples.

### 3.5. Changes in Volatile Flavor Compounds During the Cooking of Sheep Bone Soup

**Figure 3:**
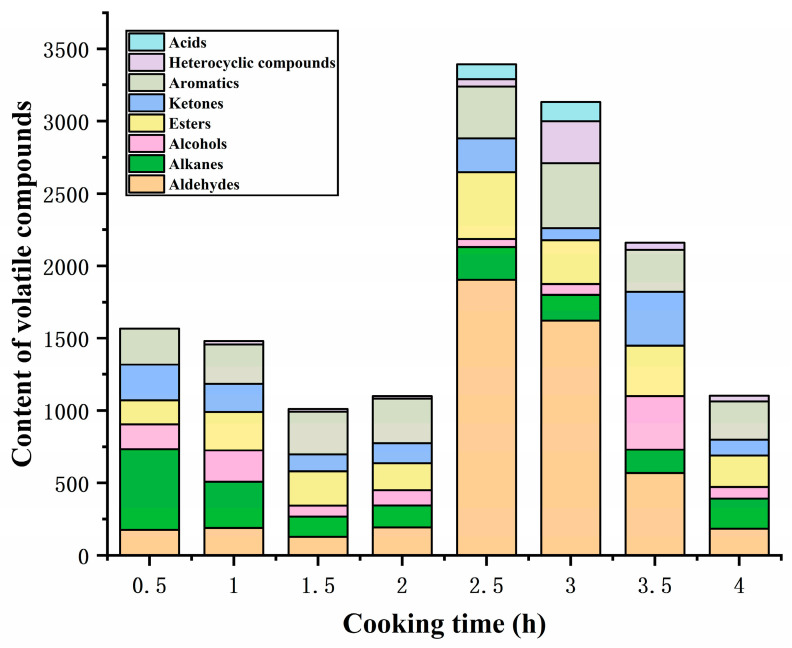
Content of volatile flavor compounds under different cooking times.

GC-MS was employed to determine the volatile flavor compounds during the cooking process of sheep bone soup. As seen in Table 3, 39 flavor compounds were identified, including 4 aldehydes, 3 ketones, 7 alcohols, 7 esters, 1 acid, 5 aromatics, 8 alkanes, and 4 heterocyclic compounds. The total content of volatile flavor compounds exhibited a fluctuating tendency, initially decreasing and subsequently increasing during the early and middle phases of cooking, attaining the peak after 2.5 h of cooking, and then decreasing continuously as the cooking time was prolonged (Figure 3), which was similar to the changes in flavor compounds reported by Meng et al. [3] in bovine soup within 6 h of stewing. Volatile flavor compounds, including aldehydes, alcohols, esters, and ketones, are mainly produced by the oxidative degradation of lipids, but continuous heating may result in loss due to their volatile properties and interactions between components; these effects determine the variation in the content of volatile aromatic compounds [6].

Aldehydes, with low odor thresholds, are important volatile flavor compounds and have also been proposed by some researchers as the source of the oily flavor of meat products [34]. The content of aldehydes in the sheep bone soup increased and then declined with the extension of cooking time, and the total content was highest at 2.5 h of cooking, as shown in Figure 3. Four straight-chain aldehydes were detected in sheep bone soup: heptanal, hexanal, pentanal, and 2-propenal, with heptanal being the most abundant. Aldehydes were derived from the oxidation of unsaturated fatty acids and Strecker degradation of amino acids [35], and linear aldehydes are considered to be most likely associated with the oxidation of unsaturated fatty acids [36]. Usually, aldehydes containing 6–9 carbon atoms are the main volatile aldehydes, which can overlap flavor with other compounds and contribute to the overall flavor profile [37,38]. Heptanal and pentanal have been described as meaty and almondy flavors, respectively, which are formed by the oxidative derivatization of oleic acid [39]. Hexanal exhibits a grassy aroma at low concentrations [40] and is formed through the degradation of oleic acid and linoleic acid [41]. The unsaturated aldehyde 2-propenal, which may have originated from the oxidation of unsaturated lipids [42], only appeared at 3 h of cooking with low content and made a minor contribution to the soup flavor. In this study, the significant increase in the content of aldehydes in sheep bone soup after 2 h of cooking was mainly ascribed to the oxidation and degradation of unsaturated fatty acids, particularly oleic acid, whose content began to decline sharply at this time. However, as carbonyl compounds, aldehydes could react with amino acids, peptides, proteins, and other substances [40], which might explain the decline in aldehyde content after 3 h of cooking.

Ketones have a much higher threshold than their isomeric aldehydes and a much smaller flavor contribution than aldehydes [43]. The ketones in sheep bone soup included acetone, 3-pentanone, and 2,3-pentanedione, and ketones can engender floral, fruity, and buttery flavors [44]. Hallier et al. [45] reported that 2,3-pentanedione provided an ideal balance of meaty and buttery flavors in European catfish. The ketone content in sheep bone soup fluctuated during the cooking process, reaching its peak at 3.5 h. Among them, the content of acetone was relatively high and showed a fluctuating downward trend throughout the cooking process. 3-pentanone and 2,3-pentanedione were mainly detected in the middle and later stages of cooking and exhibited a trend of rising first and then falling. Ketones can be produced through the thermal oxidation or degradation of unsaturated fatty acids, amino acid degradation, and the Maillard reaction [46,47,48]. Similar to aldehydes, the carbonyl groups of ketones could react with the amino groups of amino acids and proteins, causing a loss in their contents [40]. Combined with Figure 1, with prolongation of the cooking time, the ketone compounds fluctuated between generation and consumption due to the Maillard reaction and lipid oxidation. Additionally, amino acid Strecker degradation under the participation of diketone compounds can produce aminoketone or mercapto acetaldehyde, which are the precursors for the generation of sulfur-containing and nitrogen-containing heterocyclic compounds [49]. This may be one of the reasons for the low 2,3-pentanedione content in the bone soup.

Alcohols constitute a relatively significant group of flavor compounds. Seven alcohols were identified in sheep bone soup, including four straight-chain alcohols and three branched-chain alcohols. Similar to ketones, the content of alcohols underwent fluctuating changes, with the highest content attained after cooking for 3.5 h. In the early stages of cooking, alcohols were mainly branched alcohols, whereas the proportion of straight-chain alcohols increased in the middle and later stages. This may be attributed to the fact that straight-chain alcohols primarily originate from the secondary degradation of hydroperoxides generated by fatty acids oxidation, while branched alcohols are generally produced by the decomposition of branched aldehydes or Strecker degradation of amino acids [50,51]. Alcohols could further react with acids to form esters and oxidize to produce acids and other compounds, which was induced by the decline in alcohol contents. Three alcohols were found, including 2-methyl-2-propanol, 1-butanol, and 2,3-butanediol. They could present flavors such as alcohol, camphor, and sweetness due to relatively high concentrations. However, saturated alcohols contributed less significantly to flavor due to their high odor threshold. Unsaturated alcohols had a lower flavor threshold [52], but there was only one unsaturated alcohol in the bone soup, 2-propanol, which had a relatively low content and was only detected in the initial cooking stages. Therefore, the contribution of alcohols to the flavor was lower than that of aldehydes.

Seven esters were found in sheep bone soup, among which ethyl acetate was the most abundant. Ethyl acetate was identified throughout the cooking process and presented a sweet and fruity flavor. Compared to the alcohols and ketones in bone soup, esters had a lower flavor threshold [53] and a relatively higher content, contributing more to the flavor of bone soup. During the cooking process, the content of esters generally increased at first and then decreased, peaking at 2.5 h. Esters were mainly formed through esterification reactions between carboxylic acids and alcohols produced by lipid oxidation [54], and the decrease in contents was attributed to further degradation [55].

Acids are mainly derived from the oxidation of lipids and aldehydes [56]. Only one acid, 2-propenoic acid, was detected in the bone soup, which might be derived from 2-propenal by oxidation and was inclined to undergo esterification reactions to form ester substances. It was detectable only at 2.5 h and 3 h.

Aromatic compounds originate partly from the degradation of amino acids [57], and partly from volatile organic compounds in the environment. Aromatic compounds have a low threshold and thus contribute to the overall flavor [58]. According to Figure 3, the content of aromatics increased initially during the cooking process and started to decline after 3 h. Among them, the contents of toluene and naphthalene were relatively high; the former had a chemical and solvent flavor [59], while the latter had a clean and chestnut-like aroma [60]. In addition to the pyrolysis of free tyrosine, toluene can also be formed from unsaturated hydrocarbons generated through the oxidative decomposition products of fatty acids [61], with its content showing a similar trend to that of aldehydes in Table 3. During prolonged heating, the double bonds in the benzene ring might be disrupted and generate other compounds, which would lower the amount of aromatic compounds during the later stage of cooking. The only aromatic aldehyde found in sheep bone soup was 2,4-dimethyl benzaldehyde, which had a mild almond aroma and was formed by phenylalanine Strecker degradation or the linolenic acid oxidation pathway [62,63]. However, it was detected only in the initial stage of cooking and had little contribution to the flavor of the bone soup.

Alkanes contribute less to the flavor due to the high flavor threshold, but they positively impact the overall flavor as flavor coordinators [64,65]. During cooking, eight alkanes were detected, mainly hexane and alkoxy alkanes. Hexane was detected during the latter stages of cooking, whereas alkoxy alkanes were mainly detected during the early stages of cooking. Alkane compounds primarily originate from thermal homolysis or oxidative degradation of long-chain fatty acids [66], and alkoxy alkanes may be further converted into other oxygen-containing compounds during cooking.

Four heterocyclic compounds were detected in the bone soup, including oxygen-containing tetrahydrofuran and methyl oxirane, and nitrogen-containing pyrrolidine and azetidine, which exhibit a faintly sweet, ether, and seafood flavor. Heterocyclic compounds are considered to be important flavor compounds in cooked meat, mainly originating from the Maillard reaction and the degradation of sugars in the Maillard reaction and thermal degradation of amino acids [38]. Oxygen-containing heterocycles, such as furans, originate not only from the Maillard reaction and carbohydrate dehydration but also from the oxidation of fatty acids [67]. Oxygen-containing heterocycles were predominantly detected during the latter stages of the cooking process and had higher contents compared with nitrogen-containing heterocycles. Tetrahydrofuran was the only oxygen-containing heterocycle identified throughout the entire cooking process. The content of heterocyclic compounds displayed an initial increase followed by a decline due to the thermal decomposition during the cooking process, with the content reaching its peak at 3 h of cooking.

### 3.6. Principal Component Analysis

**Figure 4:**
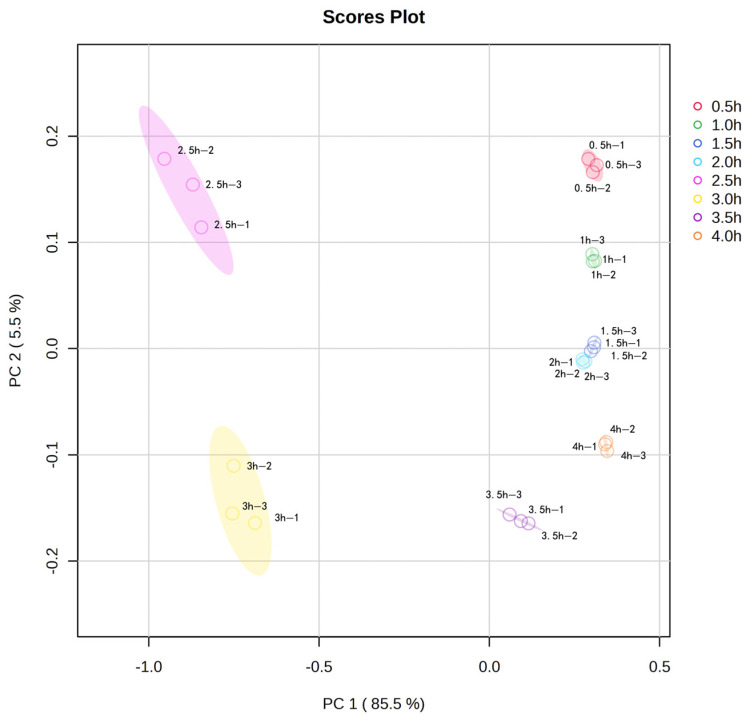
Principal component analysis (PCA) plot of volatile flavor compounds in sheep bone soup.

Principal component analysis (PCA) was performed on the volatile flavor compounds in sheep bone soup for further investigation. Figure 4 provided information on the positional distribution of the different volatile flavor compounds, and there were eight clear groups corresponding to the samples of sheep bone soup cooked for 0.5–4 h. As shown in Figure 4, the first principal component PC1 (85.5%), and the second principal component PC2 (5.5%), both with an overall contribution rate of 91.0%, were able to respond to the main information of the flavor compounds in sheep bone soup. Meanwhile, the contribution rate of PC1 was much larger than that of PC2, indicating that the first principal component can better represent the initial information of the sample. The greater the distance of the samples in the horizontal coordinate, the more obvious the variability of the samples. The samples in the 0.5 h, 1 h, 1.5 h, 2 h, and 4 h groups had a closer distribution area on the horizontal coordinate, indicating that their total flavor compositions were similar according to the distribution at each time point in the figure. However, the overall distribution area of the samples in the 2.5 h and 3 h groups was far away from the other groups, and differed significantly from other samples, so it can be seen that 2.5 and 3 h of cooking caused significant changes in the flavor of sheep bone soup.

### 3.7. Partial Least Squares Discrimination Analysis (PLS-DA) for Screening Important Volatile Flavor Compounds

**Figure 5:**
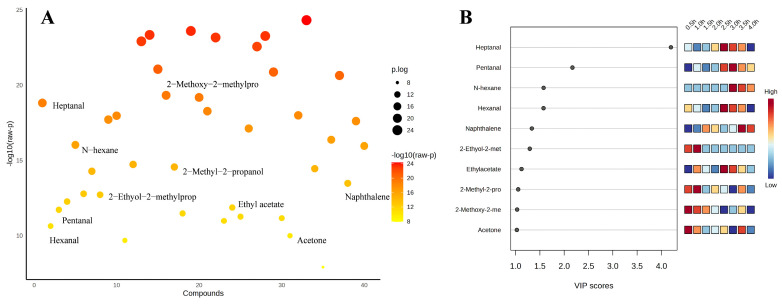
Multivariate statistical analysis results (**A**), ANOVA plot (**B**), variable importance projection of PLS-DA analysis (top 10 flavor substances with large VIP values).

Based on the PCA data, supervised discriminant analysis was continued using PLS-DA. Figure 5A displayed the analysis of variance (ANOVA) for volatile flavor compounds to analyze the differences between the sample groups. Figure 5B shows the information of the top 10 flavor compounds with large VIP values screened by PLS-DA, and then the important volatile flavor compounds of sheep bone soup were screened by the criteria of VIP > 1 and *p* < 0.05 (SPSS significance). The impact on the overall flavor increases with the VIP value of the volatile flavor compounds [68]. Ten important volatile flavor compounds were identified, including heptanal, pentanal, hexane, hexanal, naphthalene, 2-ethoxy-2-methylpropane, ethyl acetate, 2-methyl-2-propanol, 2-methoxy-2-methylpropane, and acetone. As shown in Figure 5B, 10 important volatile flavor compounds were clustered into two categories during the cooking process. The contents of 2-methyl-2-propanol, 2-methoxy-2-methylpropane, acetone, and 2-ethoxy-2-methylpropane were high during the first 2 h, and heptanal, pentanal, hexane, hexanal, naphthalene, and ethyl acetate were abundant after 2 h. Although they had high thresholds, 2-methoxy-2-methylpropane, 2-ethoxy-2-methylpropane, hexane, acetone, and 2-methyl-2-propanol might contribute to the overall flavor [69]. Aldehydes, which were most abundant at 2.5 and 3 h, accounted for approximately one-third of the total important volatile flavor compounds. Heptanal was the volatile flavor compound with the highest VIP value, which was positively correlated with its low threshold value and abundance. Ethyl acetate also contributed more to flavor due to its relatively lower flavor threshold and relatively higher content. Naphthalene had a modifying effect on the overall flavor of sheep bone soup. Continuous heating for over 2 h was conducive to the formation of aldehydes, ethyl acetate, and naphthalene, and the composition of the flavor components was also more diverse. However, if the heating duration was excessively long, exceeding 3.5 h, it was prone to causing a reactive loss of their contents.

## 4. Conclusions

During the atmospheric-pressure cooking process of sheep bone soup, the extent of lipid oxidation and Maillard reaction underwent continuous changes as the cooking time lengthened. The formation of volatile flavor compounds in sheep bone soup was closely associated with lipid oxidation, particularly the oxidation of unsaturated fatty acids and the Maillard reaction with the total content exhibiting a trend of initially rising and subsequently falling throughout the cooking process. The contents of different volatile compounds varied significantly with alterations in cooking time. Among them, aldehydes were mostly influenced by cooking time, with a notable increase in content after 2.5 h of cooking. Aldehydes were the main flavor compounds in the bone soup, with esters coming in second. The formation of flavor compounds in sheep bone soup demanded a certain amount of heating time. However, prolonged heating could lead to the loss of flavor compounds. On the whole, when the cooking time was between 2.5 h and 3 h, the contents of volatile compounds were the highest and the compositions were also relatively rich. Our study introduced in detail the volatile compounds related to the flavor of sheep bone soup, which has certain significance for improving the flavor quality of sheep bone soup. It can be seen that the concentration of volatile flavor compounds in sheep bone soup without any additives in the study is relatively low. The addition of ingredients such as salt may be used to promote the formation of flavor compounds [69], which requires further research.

## Figures and Tables

**Table 1:** Content of fatty acids in sheep bone soup under different cooking times.

No.	FA	Content (mg/kg)							
		0.5 h	1 h	1.5 h	2 h	2.5 h	3 h	3.5 h	4 h
1	C6:0	ND	ND	ND	ND	84.27 ± 2.17 ^c^	68.68 ± 8.24 ^c^	160.72 ± 5.10 ^b^	209.43 ± 26.40 ^a^
2	C8:0	67.87 ± 7.38 ^d^	72.51 ± 1.89 ^d^	138.85 ± 18.59 ^b^	200.40 ± 11.03 ^a^	109.94 ± 16.70 ^c^	62.47 ± 1.72 ^d^	148.45 ± 6.71 ^b^	76.78 ± 0.68 ^d^
3	C10:0	35.59 ± 1.94 ^f^	59.19 ± 2.52 ^de^	92.68 ± 2.75 ^c^	63.16 ± 2.41 ^d^	46.65 ± 6.70 ^ef^	45.25 ± 6.89 ^ef^	207.41 ± 8.85 ^a^	139.17 ± 16.38 ^b^
4	C11:0	79.35 ± 3.58 ^f^	156.15 ± 5.32 ^d^	128.16 ± 22.96 ^e^	206.33 ± 24.60 ^c^	97.95 ± 4.38 ^f^	76.64 ± 4.52 ^f^	595.37 ± 8.42 ^a^	367.03 ± 7.15 ^b^
5	C12:0	ND	ND	ND	ND	27.70 ± 2.36 ^c^	17.94 ± 1.01 ^d^	62.15 ± 4.42 ^a^	57.12 ± 1.70 ^b^
6	C13:0	24.15 ± 3.97 ^e^	ND	48.54 ± 2.75 ^d^	106.12 ± 8.48 ^c^	96.05 ± 7.58 ^c^	31.68 ± 1.30 ^e^	211.39 ± 11.98 ^a^	119.48 ± 3.26 b
7	C14:1,cis-9	77.89 ± 1.33 ^e^	108.32 ± 8.03 ^d^	214.68 ± 14.22 ^b^	189.97 ± 19.57 ^c^	104.77 ± 12.52 ^d^	111.34 ± 8.41 ^d^	303.39 ± 18.26 ^a^	75.88 ± 10.89 ^e^
8	C16:0	39.18 ± 5.94 ^c^	71.33 ± 8.44 ^a^	0.00 ± 0.00 ^e^	77.99 ± 6.61 ^a^	50.09 ± 7.17 ^b^	15.97 ± 2.39 ^d^	39.36 ± 2.24 ^c^	56.09 ± 0.88 ^b^
9	C16:1,cis-9	43.71 ± 0.96 ^f^	66.82 ± 1.33 ^de^	188.98 ± 1.75 ^a^	84.62 ± 3.75 ^d^	65.44 ± 5.14 ^e^	33.58 ± 0.95 ^f^	163.42 ± 22.89 ^b^	106.77 ± 19.36 ^c^
10	C18:0	161.67 ± 16.22 ^e^	989.93 ± 92.26 ^b^	475.62 ± 34.42 ^c^	1024.75 ± 56.91 ^b^	365.04 ± 22.43 ^d^	271.51 ± 14.75 ^d^	1290.28 ± 9.81 ^a^	974.29 ± 65.17 ^b^
11	C18:1,cis-9	194.73 ± 7.82 ^b^	141.47 ± 4.58 ^c^	124.70 ± 2.59 ^d^	225.44 ± 5.45 ^a^	63.17 ± 0.27 ^e^	32.49 ± 5.91 ^h^	54.17 ± 3.86 ^f^	43.47 ± 0.15 ^g^
12	C18:2,cis-9,12	23.04 ± 2.62 ^a^	ND	ND	ND	ND	ND	ND	ND
13	C22:0	74.89 ± 3.92 ^d^	ND	57.85 ± 4.72 ^d^	0.00 ± 0.00 ^e^	188.19 ± 10.39 ^c^	334.14 ± 32.56 ^b^	897.51 ± 96.25 ^a^	187.51 ± 37.16 ^c^
14	C20:3,cis8,11,14	0.00 ± 0.00 ^b^	ND	ND	ND	ND	ND	78.42 ± 0.36 ^a^	ND
	SFA	482.71 ± 16.18 ^f^	1349.10 ± 109.50 ^d^	941.69 ± 45.92 ^e^	1678.78 ± 71.03 ^c^	1065.87 ± 32.32 ^e^	924.29 ± 44.96 ^e^	3612.66 ± 66.57 ^a^	2186.89 ± 111.32 ^b^
	MUFA	316.33 ± 8.19 ^c^	316.61 ± 6.69 ^c^	528.36 ± 14.66 ^a^	500.03 ± 11.73 ^b^	233.38 ± 10.22 ^d^	177.41 ± 13.33 ^e^	520.99 ± 6.79 ^ab^	226.12 ± 19.42 ^d^
	PUFA	23.04 ± 2.62 ^b^	0.00 ± 0.00 ^c^	0.00 ± 0.00 ^c^	0.00 ± 0.00 ^c^	0.00 ± 0.00 ^c^	0.00 ± 0.00 ^e^	78.42 ± 0.35 ^a^	0.00 ± 0.00 ^c^
	TFA	822.08 ± 24.06 ^h^	1665.71 ± 114.09 ^d^	1470.06 ± 33.78 ^e^	2178.79 ± 82.73 ^c^	1299.26 ± 29.65 ^f^	1101.71 ± 38.03 ^g^	4212.07 ± 61.47 ^a^	2413.02 ± 103.03 ^b^

FA: fatty acids. SFA: saturated fatty acids. UFA: unsaturated fatty acids. TFA: total fatty acids. ^a–h^ represent significant differences (*p* < 0.05) between samples at different cooking times. ND: not detected.

**Table 2:** The performance of the E-nose sensors.

No.	Sensor	Sensitivity (mL/m^3^)	Sensitive Substances
1	W1C	10	Aromatic compounds, benzene
2	W5S	1	Nitrogen oxides
3	W3C	10	Aromatic compounds, Ammonia
4	W6S	100	Hydrocarbons
5	W5C	1	Short-chain alkanes
6	W1S	100	Methyls
7	W1W	1	Sulfides
8	W2S	100	Alcohols, aldehydes, and ketones
9	W2W	1	Aromatic compounds, organic sulfides
10	W3S	100	Long-chain alkanes

**Table 3:** Content of volatile flavor compounds in sheep bone soup under different cooking times.

Compound	RT (min)	RI	Content (ng/mL)							
			0.5 h	1 h	1.5 h	2 h	2.5 h	3 h	3.5 h	4 h
Heptanal	13.05	902.4	88.48 ± 13.18 ^d^	47.61 ± 1.63 ^de^	49.05 ± 3.53 ^de^	89.11 ± 2.24 ^d^	1052.04 ± 30.29 ^a^	978.55 ± 53.71 ^b^	239.48 ± 32.78 ^c^	0.00 ± 0.00 ^e^
Hexanal	9.96	801.9	88.74 ± 1.35 ^cd^	87.89 ± 5.49 ^cd^	55.44 ± 3.13 ^d^	60.18 ± 3.28 ^d^	659.75 ± 104.95 ^a^	396.76 ± 56.61 ^b^	146.12 ± 0.59 ^c^	38.11 ± 2.52 ^d^
Pentanal	7.25	701.7	ND	54.53 ± 9.09 ^d^	22.72 ± 3.89 ^ef^	43.37 ± 5.26 ^de^	194.15 ± 3.11 ^ab^	214.39 ± 32.03 ^a^	183.67 ± 1.73 ^b^	114.81 ± 18.12 ^c^
2-Propenal	6.93	683.3	ND	ND	ND	ND	ND	31.29 ± 0.81 ^a^	ND	0.03 ± 0.01 ^a^
Aldehydes			177.22 ± 14.54 ^d^	190.02 ± 11.34 ^d^	127.21 ± 9.26 ^e^	192.66 ± 6.00 ^d^	1905.94 ± 121.21 ^a^	1620.99 ± 28.63 ^b^	569.27 ± 33.71 ^c^	184.47 ± 25.63 ^d^
Acetone	4.45	505.5	233.66 ± 23.53 ^a^	194.15 ± 7.59 ^b^	116.00 ± 11.27 ^c^	118.18 ± 1.45 ^c^	118.89 ± 1.45 ^c^	ND	221.83 ± 30.24 ^ab^	108.86 ± 10.37 ^c^
2,3-Pentanedione	5.97	625.9	ND	ND	ND	2.53 ± 0.29 ^b^	55.44 ± 3.55 ^a^	ND	1.55 ± 0.17 b	ND
3-Pentanone	5.47	594.6	13.84 ± 0.42 ^e^	ND	ND	17.89 ± 2.10 ^d^	60.63 ± 2.06 ^c^	82.59 ± 2.53 ^b^	150.50 ± 0.72 ^a^	1.51 ± 0.37 ^f^
Ketones			246.49 ± 23.94 ^b^	194.15 ± 7.59 ^c^	116.00 ± 11.27 ^d^	138.59 ± 1.48 ^d^	234.95 ± 6.48 ^b^	82.59 ± 2.53 ^e^	373.89 ± 30.87 ^a^	110.37 ± 10.00 ^de^
2-Methyl-2-propanol	4.61	519.7	162.81 ± 15.09 ^a^	170.16 ± 0.22 ^a^	54.25 ± 3.89 ^c^	60.84 ± 5.74 ^c^	55.47 ± 5.49 ^c^	31.60 ± 0.73 ^d^	149.12 ± 3.55 ^b^	39.23 ± 1.37 ^d^
1-Butanol	6.56	660.8	ND	ND	16.27 ± 1.33 ^d^	32.52 ± 1.58 ^b^	ND	19.14 ± 1.03 ^d^	157.72 ± 2.65 ^a^	24.55 ± 2.39 ^c^
2-Propenol	16.25	1026.9	7.06 ± 0.34 ^b^	27.13 ± 4.29 ^a^	ND	ND	ND	ND	ND	ND
DL-2,3-Butanediol	6.54	659.8	ND	ND	2.62 ± 0.31 ^b^	4.23 ± 0.44 ^b^	ND	2.66 ± 0.27 ^b^	62.62 ± 3.29 ^a^	ND
4-Methyl-1-pentanol	5.91	622.4	ND	ND	ND	3.97 ± 0.10 ^c^	ND	11.78 ± 0.48 ^b^	ND	15.59 ± 0.92 ^a^
1,4-Butanediol	10.97	834.7	ND	16.28 ± 0.33 ^a^	4.10 ± 0.25 ^c^	ND	ND	9.66 ± 0.29 ^b^	ND	ND
4-Amino-1-butanol	6.92	681.9	ND	3.94 ± 0.64 ^a^	ND	2.64 ± 0.25 ^b^	ND	ND	ND	ND
Alcohols			169.87 ± 15.44 ^c^	217.52 ± 4.06 ^b^	77.24 ± 4.17 ^e^	104.19 ± 7.22 ^d^	55.47 ± 5.49 ^f^	74.85 ± 1.66 ^e^	369.46 ± 0.70 ^a^	79.38 ± 3.29 ^e^
Ethyl acetate	5.67	608.3	107.26 ± 0.01 ^f^	247.15 ± 18.73 ^c^	206.54 ± 10.91 ^d^	141.95 ± 18.88 ^e^	401.03 ± 17.81 ^a^	299.73 ± 5.34 ^b^	246.92 ± 20.54 ^c^	189.32 ± 8.28 ^d^
Isobutyl nitrite	4.69	526.8	ND	11.79 ± 1.93 ^a^	ND	ND	5.86 ± 0.34 ^b^	3.74 ± 0.19 ^c^	ND	ND
Isobutyl acetate	10.42	816.7	ND	6.03 ± 0.22 ^b^	ND	4.59 ± 0.31 ^c^	ND	ND	ND	6.65 ± 0.39 ^a^
Oxalic acid, diallyl ester	9.13	771.7	34.65 ± 2.02 ^b^	ND	ND	ND	10.22 ± 0.85 ^c^	ND	65.87 ± 0.46 ^a^	ND
Ethyl diazoacetate	5.80	616.1	ND	ND	3.74 ± 0.09 ^b^	ND	7.86 ± 0.23 ^a^	ND	0.31 ± 0.01 ^c^	ND
Diethyl carbonate	8.46	746.7	ND	ND	7.79 ± 0.34 ^b^	15.67 ± 0.57 ^a^	ND	ND	15.65 ± 0.42 ^a^	ND
Tert-Butyl N-hydroxycarbamate	8.54	749.4	24.64 ± 2.48 ^b^	ND	18.52 ± 2.65 ^c^	24.51 ± 3.34 ^b^	35.43 ± 2.19 ^a^	ND	20.52 ± 0.29 ^c^	21.50 ± 0.29 ^bc^
Esters			166.55 ± 5.48 ^f^	264.97 ± 18.09 ^d^	236.59 ± 12.59 ^de^	186.71 ± 19.95 ^f^	460.40 ± 18.60 ^a^	303.47 ± 5.43 ^c^	349.28 ± 22.11 ^b^	217.47 ± 7.97 ^e^
2-Propenoic acid	9.97	802.1	ND	ND	ND	ND	100.91 ± 9.08 ^b^	133.88 ± 0.40 ^a^	ND	ND
Acids			ND	ND	ND	ND	100.91 ± 9.08 ^b^	133.88 ± 0.40 ^a^	ND	ND
Toluene	8.91	763.4	57.93 ± 10.81 ^e^	97.34 ± 2.68 ^d^	123.64 ± 4.22 ^c^	110.89 ± 17.33 ^cd^	215.49 ± 6.29 ^b^	245.53 ± 6.11 ^a^	59.37 ± 2.91 ^e^	38.84 ± 2.80 ^f^
Ethylbenzene	12.12	871.9	15.07 ± 0.33 ^d^	21.48 ± 3.46 ^cd^	37.55 ± 4.29 ^b^	27.09 ± 3.95 ^c^	38.99 ± 2.06 ^b^	23.53 ± 2.76 ^c^	34.56 ± 3.82 ^b^	52.09 ± 4.53 ^a^
1,3-Dimethyl-benzene	12.07	870.4	45.83 ± 4.57 ^b^	ND	ND	45.35 ± 3.34 ^b^	ND	69.64 ± 0.91 ^a^	ND	ND
Naphthalene	19.42	1196.9	61.19 ± 4.31 ^e^	68.83 ± 10.61 ^e^	134.68 ± 9.63 ^c^	123.49 ± 0.73 ^c^	102.08 ± 3.72 ^d^	110.60 ± 4.00 ^d^	196.63 ± 4.14 ^a^	172.13 ± 1.89 ^b^
2,4-Dimethyl-benzaldehyde	19.88	1227.5	69.81 ± 1.35 ^b^	83.29 ± 3.43 ^a^	ND	ND	ND	ND	ND	ND
Aromatics			249.83 ± 18.74 ^e^	270.94 ± 12.19 ^de^	295.87 ± 14.14 ^c^	306.83 ± 13.99 ^c^	356.57 ± 2.61 ^b^	449.30 ± 11.89 ^a^	290.55 ± 4.67 ^cd^	263.05 ± 8.11 ^e^
Hexane	5.44	592.4	ND	ND	ND	ND	ND	149.20 ± 7.75 ^a^	130.77 ± 3.89 ^b^	107.10 ± 11.19 ^c^
2-Ethoxy-2-methylpropane	5.58	602.9	121.15 ± 5.83 ^b^	141.73 ± 18.94 ^a^	ND	ND	ND	ND	ND	ND
2-Methoxy-2-methylpropane	5.16	567.9	97.35 ± 7.51 ^a^	95.35 ± 6.35 ^a^	28.74 ± 0.91 ^b^	25.13 ± 4.42 ^b^	ND	24.09 ± 3.14 ^b^	28.43 ± 3.20 ^b^	ND
2-Ethoxy-propane	5.38	586.7	97.94 ± 5.92 ^a^	40.47 ± 2.49 ^b^	ND	15.46 ± 0.74 ^c^	ND	ND	ND	ND
1,1-Diethoxy-ethane	8.08	732.5	ND	5.98 ± 0.45 ^a^	ND	ND	4.39 ± 0.21 ^b^	6.18 ± 0.09 ^a^	0.93 ± 0.02 ^c^	ND
2-Methyl-butane	9.93	801.0	33.56 ± 2.09 ^a^	ND	13.67 ± 1.95 ^b^	31.60 ± 8.02 ^a^	ND	ND	ND	ND
2-Methoxy-pentane	5.63	606.3	35.68 ± 0.98 ^b^	34.69 ± 1.16 ^b^	ND	ND	37.49 ± 0.49 ^a^	ND	ND	4.92 ± 0.16 ^c^
Dimethyl-diazene	4.51	510.7	170.99 ± 2.88 ^b^	ND	97.49 ± 3.48 ^c^	80.71 ± 3.26 ^d^	183.42 ± 3.82 ^a^	ND	ND	95.61 ± 7.72 ^c^
Alkanes			556.68 ± 20.40 ^a^	318.21 ± 24.98 ^b^	139.89 ± 3.16 ^e^	152.90 ± 5.34 ^e^	225.31 ± 3.98 ^c^	179.47 ± 9.99 ^d^	160.12 ± 7.01 ^de^	207.63 ± 9.49 ^c^
Pyrrolidine	5.05	558.4	ND	4.87 ± 0.34 ^c^	1.55 ± 0.43 ^d^	ND	7.73 ± 0.02 ^b^	15.78 ± 0.32 ^a^	ND	ND
Azetidine	5.28	578.1	ND	ND	3.26 ± 0.12 ^c^	ND	ND	9.86 ± 0.69 ^b^	ND	14.66 ± 1.44 ^a^
Methyloxirane	4.45	506.0	ND	ND	0.00 ± 0.00 ^b^	ND	ND	215.21 ± 24.76 ^a^	ND	ND
Tetrahydrofuran	5.94	624.8	ND	18.94 ± 2.84 ^d^	13.09 ± 0.78 ^e^	16.43 ± 0.51 ^d^	44.88 ± 2.85 ^b^	47.36 ± 1.21 ^ab^	48.73 ± 0.69 ^a^	25.08 ± 0.13 ^c^
Heterocyclic compounds			ND	23.81 ± 3.03 ^cd^	17.91 ± 0.69 ^de^	16.43 ± 0.51 ^de^	52.61 ± 2.90 ^b^	288.21 ± 25.25 ^a^	48.73 ± 0.69 ^b^	39.74 ± 1.31 ^bc^
All			1566.64 ± 71.66 ^d^	1479.62 ± 28.57 ^d^	1010.71 ± 39.95 ^e^	1098.33 ± 21.89 ^e^	3392.16 ± 89.97 ^a^	3132.77 ± 18.67 ^b^	2161.29 ± 80.00 ^c^	1102.11 ± 23.12 ^e^

Values represent the mean ± SD (*n* = 3). ^a–f^ represent significant differences (*p* < 0.05) between samples at different cooking times. RI: retention index. RT: retention time. ND: not detected. RI, retention index in agreement with the literature value.

## Data Availability

The original contributions presented in this study are included in the article. Further inquiries can be directed to the corresponding author.
